# Alveolar instability caused by mechanical ventilation initially damages the nondependent normal lung

**DOI:** 10.1186/cc6122

**Published:** 2007-09-18

**Authors:** Lucio Pavone, Scott Albert, Joseph DiRocco, Louis Gatto, Gary Nieman

**Affiliations:** 1 Upstate Medical University, Department of Surgery, 750 E Adams Street, Syracuse, NY 13210 USA; 2 Department of Biology, Cortland College, P.O. Box 2000 Cortland, NY 13045 USA

## Abstract

**Background:**

Septic shock is often associated with acute respiratory distress syndrome, a serious clinical problem exacerbated by improper mechanical ventilation. Ventilator-induced lung injury (VILI) can exacerbate the lung injury caused by acute respiratory distress syndrome, significantly increasing the morbidity and mortality. In this study, we asked the following questions: what is the effect of the lung position (dependent lung versus nondependent lung) on the rate at which VILI occurs in the normal lung? Will positive end-expiratory pressure (PEEP) slow the progression of lung injury in either the dependent lung or the nondependent lung?

**Materials and methods:**

Sprague–Dawley rats (*n *= 19) were placed on mechanical ventilation, and the subpleural alveolar mechanics were measured with an *in vivo *microscope. Animals were placed in the lateral decubitus position, left lung up to measure nondependent alveolar mechanics and left lung down to film dependent alveolar mechanics. Animals were ventilated with a high peak inspiratory pressure of 45 cmH_2_O and either a low PEEP of 3 cmH_2_O or a high PEEP of 10 cmH_2_O for 90 minutes. Animals were separated into four groups based on the lung position and the amount of PEEP: Group I, dependent + low PEEP (*n *= 5); Group II, nondependent + low PEEP (*n *= 4);Group III, dependent + high PEEP (*n *= 5); and Group IV, nondependent + high PEEP (*n *= 5). Hemodynamic and lung function parameters were recorded concomitant with the filming of alveolar mechanics. Histological assessment was performed at necropsy to determine the presence of lung edema.

**Results:**

VILI occurred earliest (60 min) in Group II. Alveolar instability eventually developed in Groups I and II at 75 minutes. Alveoli in both the high PEEP groups were stable for the entire experiment. There were no significant differences in arterial PO_2 _or in the degree of edema measured histologically among experimental groups.

**Conclusion:**

This open-chest animal model demonstrates that the position of the normal lung (dependent or nondependent) plays a role on the rate of VILI.

## Introduction

Mechanical ventilation (MV) is essential in the treatment of the acute respiratory distress syndrome (ARDS), but casual MV can lead to a secondary ventilator-induced lung injury (VILI) significantly increasing the morbidity and mortality [1-3]. High tidal volume MV has been shown to significantly worsen the outcome of the critically ill patient, and reducing or eliminating VILI would greatly improve the prognosis of these patients [1,4]. One of the primary mechanisms of VILI is alveolar recruitment/derecruitment, which causes a shear stress-induced mechanical injury to the pulmonary parenchyma [5]. Alveolar instability (recruitment/derecruitment) causes a cascade of pathologic events, including a direct mechanical injury to pulmonary tissue that causes a release of cytokines that can exacerbate the systemic inflammatory response syndrome typical of ARDS [6].

ARDS is a heterogeneous injury with both normal and diseased tissue throughout the lung. A study by Schreiber and colleagues showed that large tidal volumes (20 ml/kg) can rapidly injure normal rat lungs as compared with low tidal volume ventilation (4 ml/kg) [7]. Although recent experiments have shown that improper MV can injure both diseased and normal lung tissue [3,7,8], several questions concerning the pathophysiology of VILI in the normal lung remain unanswered: are different lung regions (dependent versus nondependent) more susceptible to VILI during high-volume, high-pressure ventilation? If VILI is dependent upon the lung position, will a positive end-expiratory pressure (PEEP) be protective in all lung areas?

In the present study we addressed these questions by measuring alveolar mechanics (that is, the dynamic change in alveolar size and shape with tidal ventilation) utilizing *in vivo *microscopy in both the dependent lung and the nondependent lung. Lung injury (VILI) was determined by a change from normal, stable alveolar mechanics to highly unstable alveoli that collapse and expand with each breath [5,8-13].

Our experimental model investigated the time it took, following initiation of injurious MV, to reach a predetermined level of lung injury. This model shifted the main endpoint to the time necessary to cause lung injury with injurious MV, rather than to a predetermined endpoint of time. In our study we defined lung injury to be a 20% increase in alveolar instability. We also assessed whether the 'time to alveolar instability' could be modified with the lung position (that is, nondependent versus dependent lung regions) and with increased PEEP.

To our knowledge this is the first study to directly visualize the influence of lung position on alveolar instability caused by injurious MV. We postulated that alveolar instability would develop first in the nondependent lung, since this lung region is more compliant and should receive a larger percentage of the tidal volume as compared with the dependent lung. We postulated that instability would develop in the dependent lung, but that it would take a longer time on injurious MV for injury to develop. We postulated that PEEP would prevent the development of alveolar instability in both regions, by increasing the functional residual capacity and therefore changing the location of ventilation on the pressure volume curve.

## Methods

### Surgical preparation and ventilator settings

Adult male Sprague–Dawley rats weighing 330–600 g were anesthetized with intraperitoneal ketamine (90 mg/kg) and xylazine (10 mg/kg) at the onset of the procedure, and as needed throughout the procedure to maintain surgical anesthesia. A tracheostomy was established with a 2.5 mm pediatric endotracheal tube. Paralysis was then achieved with intravenous pancuronium (0.8 mg/kg) and the rats were placed on pressure control ventilation with 50% oxygen delivered via a Galileo ventilator (Hamilton Medical Inc., Reno, NV, USA). Baseline ventilator settings included a control pressure (*P*_control_, the pressure applied above that of PEEP during the inspiratory phase) of 14 cmH_2_O and a PEEP of 3 cmH_2_O. The respiratory rate was initially titrated to maintain a PCO_2 _of 35–45 mmHg.

Rats were then placed on zero PEEP and a midline sternotomy was performed with removal of the right third through sixth ribs. Lung volume history was standardized by generating a single inflation from zero PEEP to a peak pressure of 25 cmH_2_O at a constant rate of 3 cmH_2_O/sec (PV Tool™; Hamilton Medical Inc.).

### Blood pressure measurement and fluid resuscitation

A carotid arterial catheter was placed for blood gas analysis (model ABL5; Radiometer Inc., Copenhagen, Denmark) and for inline measurement of the mean arterial pressure (TruWave™; Baxter Healthcare Corp., Irvine, CA, USA). The internal jugular vein was cannulated for fluid and drug infusion. Fluid resuscitation was performed with a 1 cm^3 ^bolus of warmed lactated Ringer's solution when the mean arterial pressure fell below 60 mmHg.

The protocol was as follows. After surgical instrumentation, the rats remained on MV and were randomly assigned to one of four groups: Group I, dependent + low PEEP (*n *= 5), *P*_control _= 45 cmH_2_O, PEEP = 3 cmH_2_O; Group II, nondependent + low PEEP (*n *= 4), *P*_control _= 45 cmH_2_O, PEEP = 3 cmH_2_O; Group III, dependent + high PEEP (*n *= 5), *P*_control _= 45 cmH_2_O, PEEP = 10 cmH_2_O; and Group IV, nondependent + high PEEP (*n *= 5), *P*_control _= 45 cmH_2_O, PEEP = 10 cmH_2_O.

**Figure 1:**
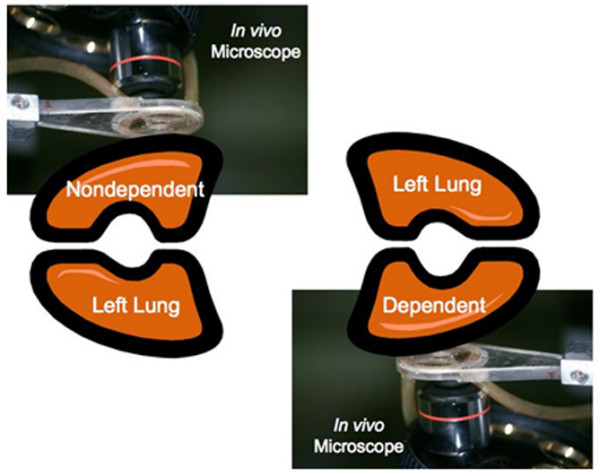
Schematic demonstrating *in vivo *videomicroscopy procedure for the nondependent and dependent lung. The right lung was filmed in all groups (that is, dependent and nondependent lung and high and low positive end-expiratory pressure). **(a) **To film the nondependent lung, the rat was placed in the left lateral decubitus position and the microscope was lowered from the top. **(b) **To film the dependent lung, the rat was in the lateral decubitus position with an open chest and the microscope was elevated from the bottom.

The only difference between the dependent and nondependent groups with similar PEEP was the position of the animal (Figure 1). Animals were placed in the lateral decubitus position, left lung up to measure the nondependent lung alveolar mechanics (Groups II and IV) and left lung down to film the dependent lung alveolar mechanics (Groups I and III) (Figure 1). *In vivo *microscopy was accomplished in the dependent lung by rotating the microscope 180° so that the objective was pointing up, and the microscope was positioned under the rat and attached to the pleural surface (Figure 1).

Concomitant with the initiation of the injurious ventilator strategy, the respiratory rate was set to 20 breaths/min in all groups. Time zero was designated as the time immediately following initiation of the experimental ventilatory strategy. Hemodynamic data, lung function data, and *in vivo *microscopic data were recorded at baseline and every 15 minutes after initiation of the experimental protocol. The protocol was terminated after 90 minutes.

### *In vivo *microscopy

**Figure 2:**
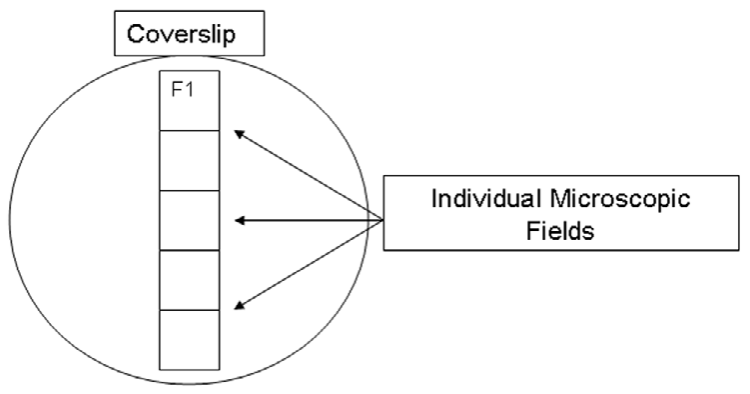
Alveolar sampling technique. The microscope objective was moved to the top of the coverslip and the first field was filmed (F1). The objective was than moved down one field, viewing all new alveoli. This was sequentially repeated to the bottom of the coverslip, filming five entirely different microscopic fields of alveoli.

A microscopic coverslip mounted on a ring was lowered onto the pleural surface, and the lung was held in place by gentle suction (≤5 cmH_2_O) at end inspiration for placement of an *in vivo *videomicroscope (epi-objective microscope with epi-illumination; Olympus America Inc. Melville, NY USA). At each timepoint, the apparatus was reattached to the lung so that a different cohort of alveoli was sampled every 15 minutes. The microscope objective was moved from the top to the bottom of the coverslip field by field, and each new field was photographed for the measurement of alveolar mechanics (Figure 2). Microscopic images of alveoli were viewed at a final magnification of 130× with a color video camera (model CCD SSC-S20; Sony, Tokyo, Japan) and recorded on Pinnacle Studio Plus software (Pagasus Imaging Corporation Tampa, FL) Each field measured 1.22 × 10^6 ^μm^2 ^and was filmed throughout five complete tidal ventilations for subsequent analysis of alveolar mechanics.

### Image analysis of alveoli

Frame-by-frame analysis was performed by capturing still images of alveoli at peak inspiration and at end expiration. For each visual field, the subset of alveoli analyzed consisted of those that contacted a vertical line bisecting the visual field and represented approximately 10 alveoli per field, the length of that line measuring approximately 1 mm. Five microscopic fields were analyzed for each animal at each timepoint (Figure 2). The outer walls of individual alveoli were manually traced at both end inspiration and end expiration. The areas of these tracings were computed with image analysis software (Empire Imaging Systems; Image Pro, Syracuse, NY, USA) and are referred to as the area at peak inspiration (*I*) and the area at end expiration (*E*). The degree of alveolar stability (%*I *- *E*Δ) was defined as the percentage decrease in alveolar size during tidal ventilation:

%*I *- *EΔ *= 100 × [(*I *- *E*)/*I*]

For each animal at each timepoint, the mean *I *and the mean *E *values were calculated, yielding a single value. These aggregate values were then used in the statistical analysis. Similarly, %*I *- *E*Δ was calculated for each individual alveolus, and the mean %*I *- *E*Δ value for each animal at each timepoint was then compared using standard statistics (see Statistical analysis).

### Lung function measurements

Arterial blood gases, systemic arterial pressures, and pulmonary parameters (tidal volume) were recorded at baseline and then at 15-minute intervals. Pulmonary parameters were measured inline by the Galileo ventilator (Hamilton Medical Inc.).

### Necropsy

The trachea was cannulated and the lung was inflated with 10% formalin by gravity to a pressure of 25 cmH_2_O. Each lung was identified as a dependent lung or a nondependent lung and was grouped for histological assessment. After 24 hours, the tissue was blocked in paraffin and serial sections were made for staining with H & E. The slides were reviewed at high magnification for edema (400×).

### Mechanism of alveolar collapse

Alveolar instability was caused in two additional rats by 30 minutes of injurious MV (peak inspiratory pressure (PIP) = 45 cmH_2_O, PEEP = 3 cmH_2_O), similar to injury in Group I and Group II of this study. This injurious ventilation caused the alveolar mechanics of subpleural alveoli to change from stable (that is, little to no change in size with ventilation) to unstable (that is, very large change is size with tidal ventilation), determined by *in vivo *microscopy within 60 minutes of application. Once unstable alveoli developed, the animals were sacrificed and the lungs were removed *en bloc *and perfused through the pulmonary artery with 10% formalin at an intravascular pressure of 20 cmH_2_O for 24 hours.

The lungs of one rat were inflated and held constant at an airway pressure of 45 cmH_2_O (when subpleural alveoli were observed to be fully inflated with the *in vivo *microscope), and the lungs of the second rat were fixed at an airway pressure of 3 cmH_2_O (when subpleural were observed to be mostly collapsed with the *in vivo *microscope). Following 24 hours of fixation at constant perfusion and airway pressure, the lungs were blocked, sliced, and stained with H & E. These data were used to identify the potential mechanism of alveolar collapse.

### Vertebrate animals

Experiments described in this study were performed in accordance with the National Institutes of Health guidelines for the use of experimental animals in research. The protocol was approved by the Committee for the Humane Use of Animals at our institution.

### Statistical analysis

All results are presented as the mean ± standard error of the mean. An all-pairs, Tukey HSD (honestly significantly different) test was used to compare more than two groups. Student's *t *test was applied for all pair-wise comparisons. We accepted *P *< 0.05 as significant. Data were analyzed using JPM software (version 5; SAS Institute Cary, NC, USA).

## Results

### Alveolar mechanics

**Figure 3:**
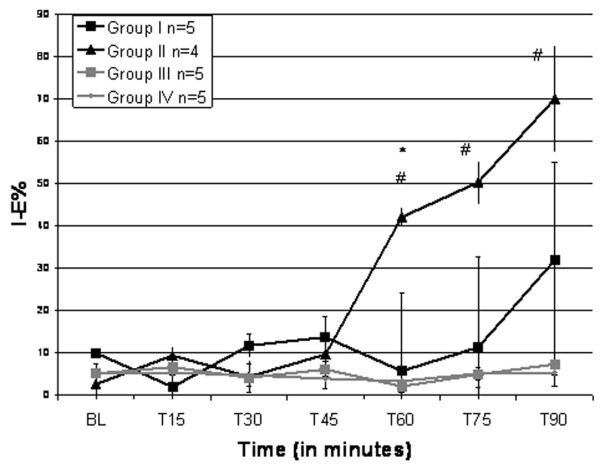
Change in alveolar stability over time. The change in alveolar stability (inspiration–expiration percentage change, %*I *- *E*) was monitored over time in four groups: Group I, dependent + low positive end-expiratory pressure (PEEP) (*n *= 5); Group II, nondependent + low PEEP (*n *= 4); Group III, dependent + high PEEP (*n *= 5); and Group IV, nondependent + high PEEP (*n *= 5). Data are the mean ± standard error. ^# ^*P *< 0.05, Group IIversus Groups III and IV; *P < 0.05, Group II versus Group I.

**Figure 4:**
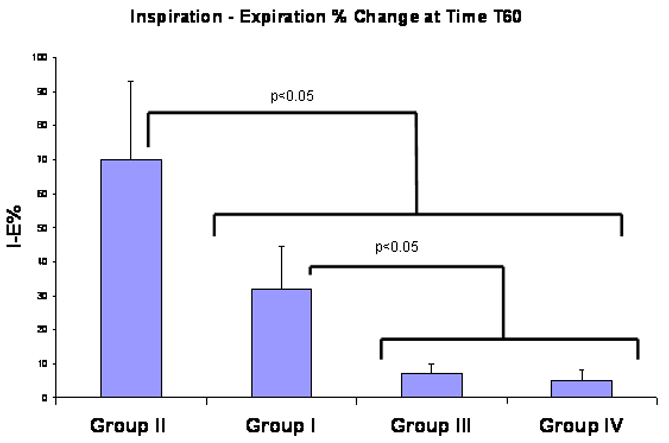
Alveolar stability at 60 minutes. The degree of alveolar stability (inspiration–expiration percentage change, %*I *- *E*) was monitored at 60 minutes in four groups: Group I, dependent + low positive end-expiratory pressure (PEEP) (*n *= 5); Group II, nondependent + low PEEP (*n *= 4); Group III, dependent + high PEEP (*n *= 5); and Group IV, nondependent + high PEEP (*n *= 5). Data are the mean ± standard error There is greatest instability in Group II, nondependent + minimal PEEP. Group III and Group IV have a PEEP of 10 cmH_2_O applied.

Normal alveoli before injurious ventilation are very stable, and they did not change size appreciably during tidal ventilation (Additional file 1). Injurious MV caused alveolar instability faster (60 minutes) in the nondependent + low PEEP group (Figure 3 and Additional file 2) as compared with the dependent + low PEEP group (Figure 3 and Additional file 3). By 75 minutes, however, the %*I *- *E*Δ was no longer different between these groups although it trended higher in the nondependent + low PEEP group. The addition of 10 cmH_2_O PEEP prevented the development of alveolar instability for the entire experiment in both the nondependent and dependent lungs (Figures 3 and 4, and Additional file 4)

### Mechanism of alveolar collapse

**Figure 5:**
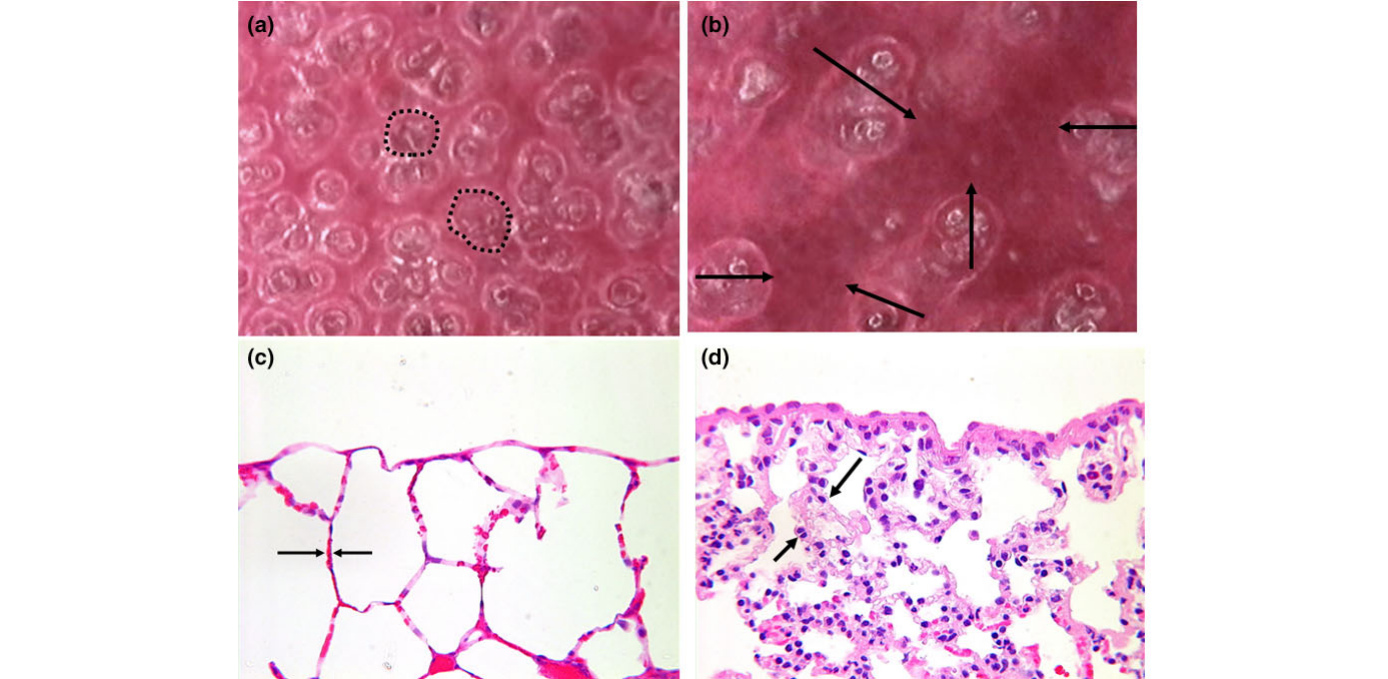
Comparison of abnormal alveoli at peak inspiration and end expiration. Abnormal alveoli at peak inspiration and end expiration as seen with an *in vivo *microscope (a, b) and as a histologic comparison (c, d). **(a) **Normal alveoli fill the microscopic field at peak inspiration, and **(b) **collapse during expiration. **(c) **Alveolar walls are very thin at peak inspiration, and **(d) **become thickened at end expiration.

At 45 cmH_2_O airway pressure (PIP) most alveoli in the *in vivo *microscopic field are inflated (Figure 5a,c), and at 3 cmH_2_O (PEEP) most alveoli collapsed (Figure 5b,d). Alveoli at the PIP are inflated and fill the *in vivo *microscopic field (Figure 5a, dotted line surrounds representative alveoli), and the alveolar walls are very thin (Figure 5c, arrows). At the PEEP the subpleural alveoli collapse (Figure 5b, dark atelectatic areas identified by arrows), and the alveolar walls are thickened (Figure 5d, arrows). The thickened alveolar walls suggest that alveolar collapse is by folding of the alveolar walls [14].

### Blood gases

The arterial PO_2 _and PCO_2 _were not significantly different in the low PEEP versus the high PEEP groups (Table 1) even though alveoli were unstable only in the low PEEP groups (Figures 3 and 4). There were no significance changes in intra-alveolar edema or in interstitial edema between groups (Table 2).

**Table 1:** Lung and hemodynamic parameters

	Baseline	15 minutes	30 minutes	45 minutes	60 minutes	75 minutes	90 minutes
Ventilation positive end-expiratory pressure 10 cmH_2_O (*n *= 10)							
PCO_2_	32.5 ± 4.40	35.7 ± 4.38	32.6 ± 4.73	31.2 ± 4.26*	28.2 ± 4.67	26.5 ± 4.21	24 ± 4.39
PO_2_	239.6 ± 15.44	293.1 ± 17.18	300.6 ± 10.66	294.5 ± 17.62	292.5 ± 21.46	331.7 ± 1.79	333.8 ± 14.23
Tidal volume (ml)	6.2 ± 0.55	3.8 ± 1.06*	3.1 ± 1.08*	3.1 ± 1.08*	2.4 ± 1.02*	2.5 ± 1.05*	2.5 ± 1.07*
Lung static compliance (ml/cmH_2_O)	0.5 ± 0.03	0.19 ± 0.03*	0.53 ± 0.29	0.47 ± 0.23	0.35 ± 0.09	0.34 ± 0.09	0.61 ± 0.34
Mean arterial pressure (mmHg)	88.5 ± 6.86	93.6 ± 14.90	87.1 ± 11.48	77.2 ± 10.75	77.8 ± 10.76	77.9 ± 10.36	58.1 ± 8.91
Fluid total^a^							9.9 ± 2.88
Ventilation positive end-expiratory pressure 3 cmH_2_O (*n *= 9)							
PCO_2_	31 ± 3.67	26.4 ± 4.27	22.5 ± 2.17	17.8 ± 1.82	17.4 ± 2.34	18 ± 2.40	17.25 ± 3.26
PO_2_	228.5 ± 24.91	293.4 ± 18.33	302.2 ± 17.62	289 ± 18.33	296.4 ± 20.85	290.78 ± 26.85	308.4 ± 32.11
Tidal volume (ml)	6.9 ± 1.53	11.5 ± 1.01	11.9 ± 1.37	12.3 ± 1.16	12.1 ± 1.18	12.9 ± 1.25	11.7 ± 1.33
Lung static compliance (ml/cmH_2_O)	0.47 ± 0.04	0.34 ± 0.02	0.32 ± 0.01	0.6 ± 0.27	0.74 ± 0.42	0.57 ± 0.25	0.53 ± 0.23
Mean arterial pressure (mmHg)	88.2 ± 8.42	78.5 ± 6.81	83.1 ± 6.81	76.4 ± 4.36	83.1 ± 9.15	82.9 ± 9.21	76.4 ± 8.12
Fluid total^a^							9.8 ± 2.74

^a^Total amount of normal saline infused over the entire experiment (ml). *P < 0.05 between groups.

**Table 2:** Pulmonary edema assessed by histological measurement of intra-alveolar edema and interstitial (alveolar wall thickness) edema

	Nondependent lung	Dependent lung
Positive end-expiratory pressure 10 cmH_2_O		
Intra-alveolar edema	3.22 ± 0.27	3.28 ± 0.25
Alveolar wall thickness	2.9 ± 0.42	2.72 ± 0.38
Positive end-expiratory pressure 3 cmH_2_O		
Intra-alveolar edema	3.5 ± 0.30	3.7 ± 0.09
Alveolar wall thickness	2.51 ± 0.45	2.62 ± 0.34

A score for both intra-alveolar and interstitial edema was used to measure edema in both nondependent and dependent lung sections: 0, no edema; 1, mild scattered edema; 2, moderate scattered edema; 3, severe scattered edema; and 4, severe universal edema. Data presented as the mean ± standard error of the mean. No significant difference was seen among groups.

### Lung function

There was a significantly smaller tidal volume in the PEEP 10 cmH_2_O groups compared with the PEEP 3 cmH_2_O groups. There was no significant difference in lung compliance or mean arterial pressure at 90 minutes between groups. There were no differences in intravenous fluid resuscitation between groups.

## Discussion

The four most important findings from this study are the following: 1) the development of alveolar injury, assessed by alveolar stability, occurred earlier following initiation of injurious ventilation in the nondependent lung with low PEEP as compared with the dependent lung with low PEEP. 2) increasing the PEEP to 10 cmH_2_O prevented alveolar instability in both the nondependent and dependent lung areas. 3) alveolar instability was not correlated with a decrease in PO_2_. 4) preventing alveolar instability with PEEP did not decrease the pulmonary edema. To our knowledge, the present study is the first to show that the position of the normal lung can influence the development of abnormal alveolar mechanics secondary to injurious MV. It is tempting to use these results and to hypothesize on the impact of the body position and VILI in humans, but extreme caution must be taken when extrapolating data from a rodent experiment into a human scenario.

Although it is beyond the scope of this paper to discuss in detail normal and abnormal alveolar mechanics (that is, the dynamic change in alveolar size and shape with tidal ventilation), it is important to understand that normal alveoli do not change size during tidal ventilation by expanding and contracting like a balloon in order to appreciate the significance of our experimental results. There are several excellent reviews on this subject [15,16] but a brief overview is as follows. The laboratory of Gil and colleagues produced the first high-quality experiments demonstrating the possibility that there may be many mechanisms by which the alveolar volume changed during ventilation [17,18]. Their experiments lead them to hypothesize that the lung volume change could be due to expansion and contraction of the alveolar ducts with little change in alveolar volume, could be due to successive alveolar recruitment/derecruitment, could be due to alveolar crumpling and uncrumpling (like a paper bag), and could be due to pleating and unpleating of alveolar corners.

More recent experiments have all demonstrated that alveoli do not expand and contract like balloons. Carney and colleagues studied lung inflation from the residual volume to 80% of the total lung capacity and found that alveoli do not change size appreciably even during large changes in lung volume; they concluded that the lung volume change is by alveolar recruitment and derecruitment [15]. These data were confirmed by Escolar and colleagues, using a sophisticated morphometric analysis, who demonstrated that there is little change in alveolar size during ventilation but there is a significant change in alveolar number [19,20].

It is also possible that the lung volume change is due to changes in the size of the alveolar mouth and duct. Kitaoka and colleagues have designed a working four-dimensional model of an alveolus and alveolar duct in which the major change in volume is due to opening and closing of the alveolar mouth [16]. The example movie (Additional file 5) demonstrates that the vast majority of the size change that occurs in a single alveolus during ventilation could be due to changes in the size of the alveolar mouth. As the size of the mouth of all alveoli comprising an air sac concomitantly open and close, the size of the alveolar duct changes size greatly; it is the expansion and contraction of the alveolar duct, not of the alveolus, that occurs during ventilation in the normal lung [16].

There is a potential artifact in our experimental technique. It is possible that the suction prevents normal pleural expansion and contraction, and thus prevents healthy alveoli from changing size normally with ventilation. There is evidence for this occurring since the pleural surface changes size to the one-third power of lung volume, and thus there must be either a change in size of or in the number of alveoli to account for this change. If this is true, than normal alveoli would be artificially stabilized and this may account for the minimal alveolar size change during tidal ventilation.

We believe, however, our microscopic technique was adequate to answer the questions we asked in this paper. We intended to demonstrate a change in alveolar mechanics from normal to abnormal, understanding that there was a potential alveolar-stabilizing artifact with our microscopic technique. Our results clearly show a dramatic change in alveolar stability from the normal to the injured, even if the microscopic preparation was preventing the full degree of alveolar volume change. The absolute changes in alveolar size may therefore not be totally accurate but the qualitative changes are very dramatic, allowing us to adequately answer our experimental question and to test our hypothesis.

In summary, normal alveoli are very stable, with changes in lung volume accommodated by normal alveolar recruitment and derecruitment and/or changes in the size of the alveolar mouth and duct. The unstable alveoli that develop 60 minutes following injurious MV are pathologic and will exacerbate the development of VILI [21]. The mechanism of this pathologic alveolar collapse and re-expansion appears to be alveolar folding and unfolding (Figure 5).

### VILI and body position

Our data are contrary to the findings of Nishimura and colleagues, who showed that lung injury was not gravity dependent [22]. Using a closed-chest rabbit VILI model they found that lung injury was not uniformly greatest in the dependent portions of the lung. Nishimura and colleagues demonstrated that lung injury was very regional but that the most severe injury always occurred in the dorsal portion of the lung regardless of whether the dorsal lung was in the dependent or nondependent position. In contrast, our study showed that the nondependent lung was the first to develop alveolar instability. Nishimura and colleagues, however, did show that prone position slowed the onset of atelectasis (VILI) [22], which supports our finding that body position affects the rate at which VILI develops.

Both of these studies suggest that VILI is not uniform throughout the lung, but rather occurs preferentially in specific areas; however, there is no consensus whether this specificity of injury is due to the gravitational or anatomical position of the lung. The reason for the discrepancy may involve the species being studied (rat versus rabbit), or the tools used to measure the injury (*in vivo *microscopy versus computed tomography scan). It is possible that there was more injury in the dorsal portions of the lung in our study, which could be not identified with *in vivo *microscopy. Likewise, there may have been a gravity-dependent increase in alveolar instability in Nishimura and colleagues' study that was not identified with the computed tomography scan. Finally, our study looked at open-chest rats whereas the Nishimura and colleagues study used closed-chest rabbits. Perhaps the influence of the chest wall resistance to inflation changed the location of injury in the two models.

In addition, the interpretation of the computed tomography scan has recently been called into question. Hubmayr suggests that the increased density seen by computed tomography scan in ARDS patients is caused by open alveoli flooded with edema rather than by atelectasis [23]. Perhaps the dorsal injury seen on the computed tomography scan occurs regardless of whether the animal is in the prone or the supine position because the anatomical shape of the rabbit lung causes increased edema in that dorsal portion of the lung.

### Alveolar instability and lung position

The lung can be described as an elastic sponge that is compressed by its own weight, especially when edematous (that is, nondependent lung compresses dependent lung), and by the weight of other organs (that is, the heart). Albert and Hubmayr [24] confirmed by computed tomography scan in humans that the heart compresses a significant amount of lung tissue and that the prone position relieves much of this compression. The weight of the nondependent lung and the heart would cause the dependent lung to become less compliant and would divert a larger percentage of the tidal volume into the more compliant nondependent lung. Veldhuizen and colleagues have previously shown that large tidal volumes cause pulmonary surfactant dysfunction [25,26]. The development of alveolar instability in our VILI model was therefore probably due to a large tidal volume-induced surfactant deactivation. In addition, if a larger tidal volume was being delivered to the more compliant nondependent lung, surfactant deactivation would be exacerbated – which may explain why alveolar instability occurred more rapidly in the nondependent lung.

These findings have clinical significance since the amount of healthy lung tissue is drastically reduced in ARDS [27], and thus a 'normal' tidal volume might direct excessively large volumes into the healthy tissue and cause VILI similar to that in the present study. Indeed, it has been shown that smaller tidal volumes significantly reduce mortality in ARDS patients [1].

### Alveolar instability and PEEP

In this study, the addition of PEEP prevented repetitive recruitment and derecruitment in both the nondependent and dependent lung regions. Our study used a PEEP of 10 cmH_2_O, since it was previously shown in our laboratory by Halter and colleagues that 10 cmH_2_O PEEP stabilized alveoli following a recruitment maneuver [13]. These data support those of Dreyfuss and colleagues that PEEP will reduce injury to the normal lung ventilated with high volumes and peak pressures [2,28]. Therefore it appears that it is not the high PIP that causes VILI, but rather the large change in pressure from PIP to the end-expiratory pressure that causes injury that ultimately results in altered alveolar mechanics.

The mechanisms by which PEEP reduces VILI and stabilizes alveoli are twofold: the increase in end-expiratory pressure could prevent alveolar collapse, or the decreased tidal volume when 10 cmH_2_O PEEP was applied could prevent alveolar overdistension. Although either mechanism could be responsible for the results in this paper, the literature supports the concept of a large tidal volume-induced deactivation of pulmonary surfactant causing alveolar instability [29]. We therefore conclude that the most probable mechanism of PEEP-induced alveolar stabilization is by prevention of alveolar collapse.

Our results are complex, however, since high PEEP prevented alveolar instability but did not reduce pulmonary edema measured histologically. This suggests that PEEP prevents the onset of mechanical VILI (that is, unstable alveoli) but not inflammatory VILI (that is, injury secondary to sequestered neutrophils). Neutrophil-released proteases and reactive oxygen species could cause an increase in vascular permeability with resultant edema formation without alveolar instability. It is possible that if we had allowed the study to continue past 90 minutes, the combination of mechanical and inflammatory injury in the low PEEP group would have caused more edema than that in the lung with high PEEP and stable alveoli. Another explanation for the increase in edema with high PEEP possibility is that barotrauma occurred in the absence of alveolar instability due to the high peak inflation pressure.

### Mechanism of alveolar collapse

Lung histology was studied at the PIP and at the PEEP to determine a potential mechanism of abnormal alveolar collapse and re-expansion. We used the histological configuration of the collapsed alveoli to speculate on the mechanism of this collapse. Tschumperlin and colleagues found that the alveolar walls were thickened at low airway pressure [14], very similar to those in the present study fixed at 3 cmH_2_O (Figure 5c, arrows). Using electron microscopy they demonstrated that the thickened alveolar walls were due to alveolar wall folding, and concluded that alveoli do not change size by balloon-like expansion and contraction but rather by folding and unfolding like a paper bag [14]. We conclude that the probable mechanism by which unstable alveoli collapse and expand in the injured lung is not by balloon-like isotropic expansion, but rather due to the folding of the alveolar walls.

### Alveolar instability and arterial PO_2_

Another interesting finding was that the arterial PO_2 _was not significantly reduced (actually it was slightly higher) in the low PEEP group with abnormal, unstable alveolar as compared with that in the high PEEP ventilation group with normal, stable alveoli.

The present study clearly demonstrated that alveoli in the low PEEP group were unstable, and we know from previous studies that alveolar instability leads to VILI if alveoli are unstable for 3–4 hours [5,12]. A normal arterial PO_2 _does not therefore necessarily identify a healthy lung with normal alveolar mechanics, and nor does it identify a lung that is not being subjected to mechanical VILI.

We postulate that the arterial PO_2 _remained elevated in our study even with unstable alveoli because oxygen was exchanged during the portion of the ventilatory cycle in which the unstable alveoli are inflated. This hypothesis was supported by Pfeiffer and colleagues, who demonstrated a cyclic change in arterial PO_2 _utilizing an ultrafast inline PO_2 _sensor [11]. The arterial PO_2 _in these studies fluctuated with each breath in an animal ARDS model with unstable alveoli. The arterial PO_2 _can therefore be maintained if the PIP is high enough to open most of the alveoli during inflation. Forcing collapsed alveoli open to improve the PO_2_, however, will greatly increase lung injury since alveolar recruitment/derecruitment is one of the primary mechanisms of VILI. These data can loosely be extrapolated to the bedside, and would suggest that it might be possible to normalize PO_2 _by increasing the airway pressure, but at the expense of causing a significant VILI.

### Critique of methodology

Our microscope has a limited depth of field (70 μm), and therefore only allows for alveolar analysis in two dimensions. Also, the subpleural alveolar mechanics might still differ from those within the lung parenchyma. Subpleural alveoli have less structural support since these alveoli are not surrounded on all sides by adjacent alveoli (that is, one wall of a subpleural alveolus is attached to the visceral pleura rather than to another alveolus). This anatomic arrangement may lessen the structural support provided by alveolar interdependence, causing subpleural alveoli to become unstable sooner than those within the lung. A classic paper by Mead and colleagues showed that even if not surrounded by alveoli on all sides, there is still a significant structural interdependence between alveoli [30].

The suction that stabilizes the lung tissue on the cover slip might prevent normal pleural expansion and contraction, and thus may prevent healthy alveoli from changing size normally with ventilation. Although we have not totally eliminated this possibility, we have shown in a previous study that suction slightly but significantly increased both the alveolar size and stability. These changes were very subtle, with an alveolar size change from expiration to inspiration being 1.1% in the suction group increasing to 8.3% in the nonsuction group [21]. This slight change in alveolar size with ventilation even without suction was in stark contrast to the dramatic change in alveolar size (for example, total collapse at end expiration or 100% change in size) that occurred following prolonged exposure to injurious MV. Suction therefore does not seem to artificially stabilize normal alveoli nor does it prevent alveoli from becoming unstable following injury.

Finally, the fact that we must open the chest to obtain our *in vivo *microscopy may alter the way that normal and injured alveoli behave mechanically.

## Conclusion

Injurious MV, over time, will cause damage to pulmonary alveoli, significantly altering their mechanics of ventilation. The mechanism of injury is probably a combination of tissue damage leading to alveolar flooding and deactivation of pulmonary surfactant by both direct mechanisms (large tidal volumes have been shown to deactivate surfactant) and indirect mechanisms (surfactant being washed off of the alveolar surface by edema fluid and deactivated by plasma proteins). Surfactant loss results in alveolar instability during ventilation. In the present study we demonstrated that the body position affects the timing of injurious MV-induced alveolar instability. We postulate that the normal dependent lung was less compliant than the nondependent lung, and thus received a smaller percentage of the total tidal volume; the larger tidal volume delivered to the nondependent lung was the cause of a more rapid injury (that is, alveolar instability). These data support the concept of volutrauma occurring in normal areas of the heterogeneously injured lung of ARDS patients. The arterial PO_2 _is not a good indicator of alveolar stability, and thus the PO_2 _alone would not be appropriate to identify protective MV strategies.

## Key messages

• Nondependent regions of the normal lung are the first to develop alveolar instability when ventilated with high PIP and low PEEP.

• Alveolar instability occurs without significant differences in lung edema.

• The addition of PEEP prevents high peak-pressure-induced alveolar instability but not the increase in pulmonary edema.

• Oxygenation is not an effective indicator of alveolar instability or of VILI.

## Abbreviations

ARDS = acute respiratory distress syndrome; H & E = hematoxylin and eosin; %*I *- *E*Δ = percentage change in alveolar area; MV = mechanical ventilation; PCO_2 _= partial pressure of carbon dioxide; *P*_control _= control pressure; PEEP = positive end expiratory pressure; PIP = peak inspiratory pressure; PO_2 _= partial pressure of oxygen; VILI = ventilator-induced lung injury.

## Competing interests

The authors declare that they have no competing interests.

## Authors' contributions

LP conducted the experiments, and analyzed and graphed the data. SA contributed to manuscript writing and editing, and to data analysis. JD assisted LP in conducting the experiments and analyzing the data. LG contributed to the experimental design, data analysis and interpretation, and performed the histologic analysis. GN contributed to the design and development of the protocol, to data analysis and interpretation, and to writing of the manuscript.

## Supplementary Material

Additional file 1 A Windows media player file containing a movie showing normal alveoli ventilated at a *P*_control _of 14 cmH_2_O and a PEEP of 3 cmH_2_O. Individual alveoli fill the microscopic field and do not change size appreciably with ventilation. Note the blood flowing around and over the alveoli. Click here for file

Additional file 2 A Windows media player file containing a movie showing alveolar instability in the nondependent low PEEP group 60 minutes following injurious ventilation. At end expiration there is a great deal of atelectasis, which appears as dark-red areas without the presence of alveolar structures. During inspiration, the collapsed alveoli reach the critical opening pressure and 'pop' open. When the critical closing pressure is reached during exhalation, the alveoli collapse. The mechanism of this collapse and re-expansion appears to be by alveolar folding and unfolding (Figure 5). Click here for file

Additional file 3 A Windows media player file containing a movie showing that alveoli are stable and appear normal (Additional file 1) in the dependent lung with low PEEP 60 minutes following injurious ventilation. Click here for file

Additional file 4 A Windows media player file containing a movie showing that alveoli are stable and appear normal (Additional file 1) with a high PEEP 90 minutes following injurious ventilation. Click here for file

Additional file 5 A Windows media player file containing a movie showing a computer-assisted design rendition of the three-dimensional changes in alveolar volume over time (addition of the time element creates a four-dimensional representation). The alveolar mouth is highlighted in red. Note the large change in the size of the mouth and the minimal changes in the size of the other portions of the alveolus. When functioning together in an air sac, the change in alveolar mouth size results in a large change in the size of the alveolar duct [16]. Click here for file

## References

[B1] Acute Respiratory Distress Syndrome Network (2000). Ventilation with lower tidal volumes as compared with traditional tidal volumes for acute lung injury and the acute respiratory distress syndrome. N Engl J Med.

[B2] Dreyfuss D, Saumon G (1998). Ventilator-induced lung injury: lessons from experimental studies. Am J Respir Crit Care Med.

[B3] Gajic O, Dara SI, Mendez JL, Adesanya AO, Festic E, Caples SM, Rana R, St Sauver JL, Lymp JF, Afessa B, Hubmayr RD (2004). Ventilator-associated lung injury in patients without acute lung injury at the onset of mechanical ventilation. Crit Care Med.

[B4] Rubenfeld GD, Caldwell E, Peabody E, Weaver J, Martin DP, Neff M, Stern EJ, Hudson LD (2005). Incidence and outcomes of acute lung injury. N Engl J Med.

[B5] Steinberg JM, Schiller HJ, Halter JM, Gatto LA, Lee HM, Pavone LA, Nieman GF (2004). Alveolar instability causes early ventilator-induced lung injury independent of neutrophils. Am J Respir Crit Care Med.

[B6] Gattinoni L, Carlesso E, Cadringher P, Valenza F, Vagginelli F, Chiumello D (2003). Physical and biological triggers of ventilator-induced lung injury and its prevention. Eur Respir J.

[B7] Schreiber T, Niemann C, Schmidt B, Karzai W (2006). A novel model of selective lung ventilation to investigate the long-term effects of ventilation-induced lung injury. Shock.

[B8] Su F, Nguyen ND, Creteur J, Cai Y, Nagy N, Anh-Dung H, Amaral A, Bruzzi de Carvalho F, Chochrad D, Vincent JL (2004). Use of low tidal volume in septic shock may decrease severity of subsequent acute lung injury. Shock.

[B9] Carney D, DiRocco J, Nieman G (2005). Dynamic alveolar mechanics and ventilator-induced lung injury. Crit Care Med.

[B10] DiRocco JD, Pavone LA, Carney DE, Lutz CJ, Gatto LA, Landas SK, Nieman GF (2006). Dynamic alveolar mechanics in four models of lung injury. Intensive Care Med.

[B11] Pfeiffer B, Syring RS, Markstaller K, Otto CM, Baumgardner JE (2006). The implications of arterial PO_2 _oscillations for conventional arterial blood gas analysis. Anesth Analg.

[B12] Halter JM, Steinberg JM, Gatto LA, Dirocco JD, Pavone LA, Schiller HJ, Albert S, Lee HM, Carney DE, Nieman GF (2007). Effect of positive end-expiratory pressure and tidal volume on alveolar instability-induced lung injury. Crit Care.

[B13] Halter JM, Steinberg JM, Schiller HJ, DaSilva M, Gatto LA, Landas S, Nieman GF (2003). Positive end-expiratory pressure after a recruitment maneuver prevents both alveolar collapse and recruitment/derecruitment. Am J Respir Crit Care Med.

[B14] Tschumperlin DJ, Margulies SS (1999). Alveolar epithelial surface area–volume relationship in isolated rat lungs. J Appl Physiol.

[B15] Carney DE, Bredenberg CE, Schiller HJ, Picone AL, McCann UG, Gatto LA, Bailey G, Fillinger M, Nieman GF (1999). The mechanism of lung volume change during mechanical ventilation. Am J Respir Crit Care Med.

[B16] Kitaoka H, Nieman GF, Fujino Y, Carney D, Dirocco J, Kawase I (2007). A 4-dimensional model of the alveolar structure. J Physiol Sci.

[B17] Gil J, Bachofen H, Gehr P, Weibel ER (1979). Alveolar volume–surface area relation in air- and saline-filled lungs fixed by vascular perfusion. J Appl Physiol.

[B18] Gil J, Weibel ER (1972). Morphological study of pressure-volume hysteresis in rat lungs fixed by vascular perfusion. Respir Physiol.

[B19] Escolar JD, Escolar A (2004). Lung hysteresis: a morphological view. Histol Histopathol.

[B20] Escolar JD, Escolar MA, Guzman J, Roques M (2003). Morphological hysteresis of the small airways. Histol Histopathol.

[B21] Pavone LA, Albert S, Carney D, Gatto LA, Halter JM, Nieman GF (2007). Injurious mechanical ventilation in the normal lung causes a progressive pathologic change in dynamic alveolar mechanics. Crit Care.

[B22] Nishimura M, Honda O, Tomiyama N, Johkoh T, Kagawa K, Nishida T (2000). Body position does not influence the location of ventilator-induced lung injury. Intensive Care Med.

[B23] Hubmayr RD (2002). Perspective on lung injury and recruitment: a skeptical look at the opening and collapse story. Am J Respir Crit Care Med.

[B24] Albert RK, Hubmayr RD (2000). The prone position eliminates compression of the lungs by the heart. Am J Respir Crit Care Med.

[B25] Veldhuizen RA, Tremblay LN, Govindarajan A, van Rozendaal BA, Haagsman HP, Slutsky AS (2000). Pulmonary surfactant is altered during mechanical ventilation of isolated rat lung. Crit Care Med.

[B26] Veldhuizen RA, Welk B, Harbottle R, Hearn S, Nag K, Petersen N, Possmayer F (2002). Mechanical ventilation of isolated rat lungs changes the structure and biophysical properties of surfactant. J Appl Physiol.

[B27] Gattinoni L, Pesenti A (2005). The concept of 'baby lung'. Intensive Care Med.

[B28] Dreyfuss D, Soler P, Basset G, Saumon G (1988). High inflation pressure pulmonary edema. Respective effects of high airway pressure, high tidal volume, and positive end-expiratory pressure. Am Rev Respir Dis.

[B29] Verbrugge SJ, Bohm SH, Gommers D, Zimmerman LJ, Lachmann B (1998). Surfactant impairment after mechanical ventilation with large alveolar surface area changes and effects of positive end-expiratory pressure. Br J Anaesth.

[B30] Mead J, Takishima T, Leith D (1970). Stress distribution in lungs: a model of pulmonary elasticity. J Appl Physiol.

